# Self-Assembly of Homo-Polyarylene Ether Into Reactive Matrix for Fabrication of Hybrid Functional Microparticles

**DOI:** 10.3389/fchem.2022.957853

**Published:** 2022-07-11

**Authors:** Du Gong, Dawei Zhang, Xiaoying Zhang, Xiaohong He, Yao Ji, Kun Jia

**Affiliations:** ^1^ Sichuan Aerospace Chuannan Initiating Explosive Technology Limited, Luzhou, China; ^2^ School of Materials and Energy, University of Electronic Science and Technology of China (UESTC), Chengdu, China; ^3^ Institute of Electronic and Information Engineering of UESTC in Guangdong, Dongguan, China

**Keywords:** polyarylene ether, self-assembly, quantum dots, microparticles, luminescence

## Abstract

Emulsion confinement self-assembly of block copolymer has witnessed increasing research interest in the recent decade, but the post-functionalization and application of the resultant polymeric micro/nano-particles are still in their infancy. In this work, a super-engineering polyarylene ether containing pendent nitrile and carboxyl (PAE-NC) has been synthesized and converted into polymeric microparticles for macromolecular enrichment *via* emulsion confinement self-assembly and subsequent surface modification. Moreover, the encapsulation capacity of PAE-NC was evaluated using hydrophobic fluorescent quantum dots (QD) as a functional probe. Particularly, we found that both the as-synthesized PAE-NC and its hydrolyzed derivatives could be converted into microparticles *via* emulsion confinement self-assembly. Furthermore, the co-self-assembly of red-emitting QD and PAE-NC enables the phase transfer of hydrophobic QD into hydrophilic luminescent microparticles with the persisted fluorescence emission. Based on these results, the current PAE-NC would be served as a versatile and robust matrix to fabricate advanced microparticles or microcapsules for various applications.

## 1 Introduction

Polymeric microparticles or microcapsules have been widely used as an effective matrix to fabricate various advanced functional materials or devices for energetic, environmental, catalytic, and biomedical applications (Chen et al., 2019; Schofield et al., 2020; Özkayalar et al., 2020; Jia et al., 2022; Li et al., 2022). Basically, the fine morphology and surface reactivity of these microparticles or microcapsules are of great importance for their practical applications (Ahangaran et al., 2019). For instance, due to their higher specific surface area, the mesoporous polymeric microparticles exhibited better adsorption and catalytic performance than their counterparts of macroporous and solid microparticles (Peng et al., 2018; Zheng et al., 2019). In addition, the immobilization of bio-reactive components (antibody, enzyme, and nucleotides) on polymeric microparticles is an indispensable step for their application in various biomedical fields (Wang et al., 2014; Daly et al., 2020; Jo and Lee, 2020; Tobias et al., 2021). Although the polymeric microparticles prepared by the classical emulsion or suspension polymerization normally exhibit uniform size distribution and tunable morphology, it is still quite challenging to prepare polymer-inorganic hybrid microparticles *via* the conventional radical chain polymerization as the involved reactive species are prone to be quenched by metal ions or salts from inorganic precursors.

The self-assembly of amphiphilic block copolymers (BCP) is regarded as a preferred methodology to prepare polymeric-inorganic hybrid microparticles; the fine morphology and surface reactivity of the final product can be readily modulated *via* tuning of interface reaction between BCP matrix and inorganic precursors or nanoparticles during the self-assembly process (Yang et al., 2016; Yan et al., 2019; Jia et al., 2020). Among various self-assembly protocols, the recently developed emulsion confinement self-assembly is emerging as a powerful methodology to fabricate functional microparticles, because the introduced emulsion droplet interface would serve as an additional parameter to tune the self-assembly kinetics (Li et al., 2015; Lee et al., 2019; Shin et al., 2020). Therefore, ranges of polymeric micro/nano-structures with special morphology that are hardly obtained *via* conventional solvent exchange–induced self-assembly have been prepared *via* the emulsion confinement self-assembly. However, the wider application of emulsion confinement self-assembly, especially in large-scale production, is still limited by the tedious chemical synthesis of involved block copolymers. In addition, although there are plenty of published works dealing with the morphology modulation of polymeric microparticles by tuning block copolymer backbones structures, surfactants compositions, and concentrations (Deng et al., 2016; Shin et al., 2018; Ku et al., 2019; Hu et al., 2021; Xu et al., 2021), the research work involving the post-modification and further application of these polymeric microparticles is still quite limited.

Herein, we discovered that the polymeric microparticles with tunable surface morphology could be obtained from the emulsion confinement self-assembly of a homopolymer, which was super-engineering polyarylene ether containing pendent nitrile and carboxyl (PAE-NC). It should be observed that the synthesis of PAE-NC can be easily scaled up *via* industrial polycondensation polymerization. Furthermore, we found that the polymeric microparticles obtained from emulsion self-assembly of PAE-NC exhibited exterior carboxyl groups, which can be employed as reactive sites for immobilization of molecular recognition agents to prepare functional beads. More interestingly, we also found that the hydrophobic fluorescent quantum dots can be firmly embedded in the PAE-NC microcapsules, leading to the water-dispersible fluorescent beads. Considering the scalable synthesis of PAE-NC matrix, post-functionalization capacity, and stable encapsulation of susceptive functional inorganic nanoparticles, the current work would open a new way for the fabrication of advanced polymeric-inorganic hybrid microparticles.

## 2 Materials and Methods

### 2.1 Materials

N-methyl pyrrolidone (NMP), dichloromethane (DCM), tetrahydrofuran (THF), hydrochloric acid, toluene, ethanol, and potassium carbonate (K_2_CO_3_) were purchased from Chengdu Chron Chemicals. Phenolphthalein (PPL), 2, 6-difluorobenzonitrile (DFBN), and sodium dodecyl sulfonate (SDS) were received from Sigma Aldrich. Core-shell quantum dots (QD, ZnCdSe@ZnS) were obtained from Wuhan Jiayuan Quantum Dots Co., Ltd. Agarose beads (AG Beads), protein A, protease inhibitor, RIPA lysate buffer, PBS powder, and SDS-PAGE 2× loading buffer were purchased from Sangon Biotech (Shanghai) Co., Ltd. All chemicals were used as obtained without further treatment.

### 2.2 Synthesis of Pendent Nitrile and Carboxyl Homopolymer

PAE-NC homopolymer was synthesized *via* the nucleophilic substitution polycondensation between PPL and DFBN. Specifically, 60 mmol PPL, 90 mmol K_2_CO_3_ (used as the catalyst), and 60.6 mmol DFBN were introduced into a three-necked flask containing 36 ml NMP and 12 ml toluene. Next, the mixtures were refluxed at 145°C for 3 h to finish the first stage and to remove the by-product of H_2_O. Then, the reaction mixture was gradually heated to 175°C and maintained for another 4 h to complete the polymerization, followed by pouring into hydrochloric acid solution (3 wt%) for 24 h to remove the excess K_2_CO_3_. Furthermore, the crude products were pulverized completely, washed by refluxing in ethanol, ddH_2_O, and dried at 80°C overnight to obtain purified powders. The number average and average molecular weight of PAE-NC were tested by the gel permeation chromatography (GPC) as 51,781 and 90,413, respectively. The as-synthesized PAE-NC was dispersed in 1 M NaOH solution and reacted at 80°C at different times to obtain the hydrolyzed PAE-NC derivatives.

### 2.3 Preparation of Polymeric Microparticles and QD@Pendent Nitrile and Carboxyl Composite Microparticles

Pristine polymeric microparticles were prepared by using as-synthesized PAE-NC or hydrolyzed derivatives [denoted as PAE-NC-Hx, where x represents hydrolysis time (h)]. In a typical process, 5 mg purified PAE-NC or PAE-NC-H1 were dissolved in 1 ml mixed solvent (100 μL THF and 900 μL DCM) and then injected into 10 ml aqueous solution (3 mg/ml) of surfactant (SDS). Next, the mixtures were emulsified with continuous stirring at room temperature for 6 h, followed by evaporation of the organic solvent, centrifuging, and washing with deionized water to obtain pristine polymeric microparticles. The QD@PAE-NC–composited microparticles were prepared using a similar protocol, except that the involved DCM contained 0.2 mg red-emitting ZnCdSe@ZnS QD. For the comparison, the classical solvent exchange–induced self-assembly was conducted by adding 1 ml PAE-NC DMF solution (5 mg/ml) into mixed solvents of ethanol (8 ml) and toluene (4 ml), followed by sonication at room temperature to obtain small-sized nanoparticles.

### 2.4 Preparation of Bio-Functionalized Polymer Beads for Antibody Enrichment

The carboxylated polymer beads obtained from the emulsion confinement self-assembly of PAE-NC-H1 were used as a matrix for the preparation of bio-functionalized beads. Specifically, the initially prepared polymer beads were dispersed in PBS buffer (0.1 M, pH = 7.4) and incubated with 2 ml EDC/PBS (10 mg/ml) at a 27°C incubator shaker for 20 min to activate exterior carboxyl groups. Next, activated beads were washed by PBS buffer three times and re-suspended in 2 ml PBS buffer containing 200 μg protein A and incubated at 37°C for 4 h to obtain protein A modified particles (abbreviated as AP beads). Finally, the AP beads were washed with PBST buffer (PBS with 0.05 wt% Tween-20) and suspended in 1 ml PBST solution at 4°C for further use. The antibody enrichment experiments were verified *via* the conventional Western blot (WB) and Coomassie brilliant blue staining (CBB) assays. Specifically, 293T cells (1×10^6^) were lysed in RIPA lysis buffer for 1 h and followed by centrifuging at 10,000 g for 10 min at 4°C. Then, the protein concentration of the supernatant was verified using a BCA protein assay kit (Thermo Fisher) and diluted to 300 μg total proteins per 500 μL lysate in a 1.5 ml EP tube. Next, 2 μL primary antibodies (Ab1) of LHX2 (diluted to 1/1000, Rabbit, Thermo Fisher) were added to the EP tube and incubated at 4°C overnight. Furthermore, 100 μL suspended AP beads (0, 2, 6, 12 h) or 20 μL commercialized protein A agarose beads (AG beads, in lysate with protease inhibitor) were added to the above EP tubes and incubated at 4°C for 2 h, followed by centrifugation and washing twice with 4°C RIPA lysate buffer (containing protease inhibitor and 0.05% Tween 20) to collect the immunoprecipitate. Finally, the immunoprecipitate was suspended 1:1 (volume ratio) in 23 μL 2×SDS-PAGE loading buffer and denatured at 100°C for 10 min followed by centrifuging to collect supernatant onto a new EP tube for further WB and CBB analysis.

### 2.5 Characterization

The chemical structures of synthesized PAE-NC and hydrolyzed derivatives were confirmed by FTIR (Shimadzu 8400S, KBr) and 1H NMR spectra (Bruker AMX-400, relative to DMSO-d6, 400 MHz), while their thermal properties were characterized by a differential scanning calorimeter (DSC, Q100, TA) and a thermogravimetric analyzer (TGA, Q50, TA), respectively. The surface morphology of the prepared microparticles and beads was observed using a scanning electron microscope (SEM, JEOL, JSM 6490LV). The fluorescent emission spectra of QD@PAE-NC and QD@PAE-NC-Hx composite microparticles were recorded with a fluorescent spectrophotometer (F-97pro, China). The luminescence images of the microparticles suspension were captured by a smartphone camera in a dark box ultraviolet analyzer.

## 3 Results and Discussion

**FIGURE 1:**
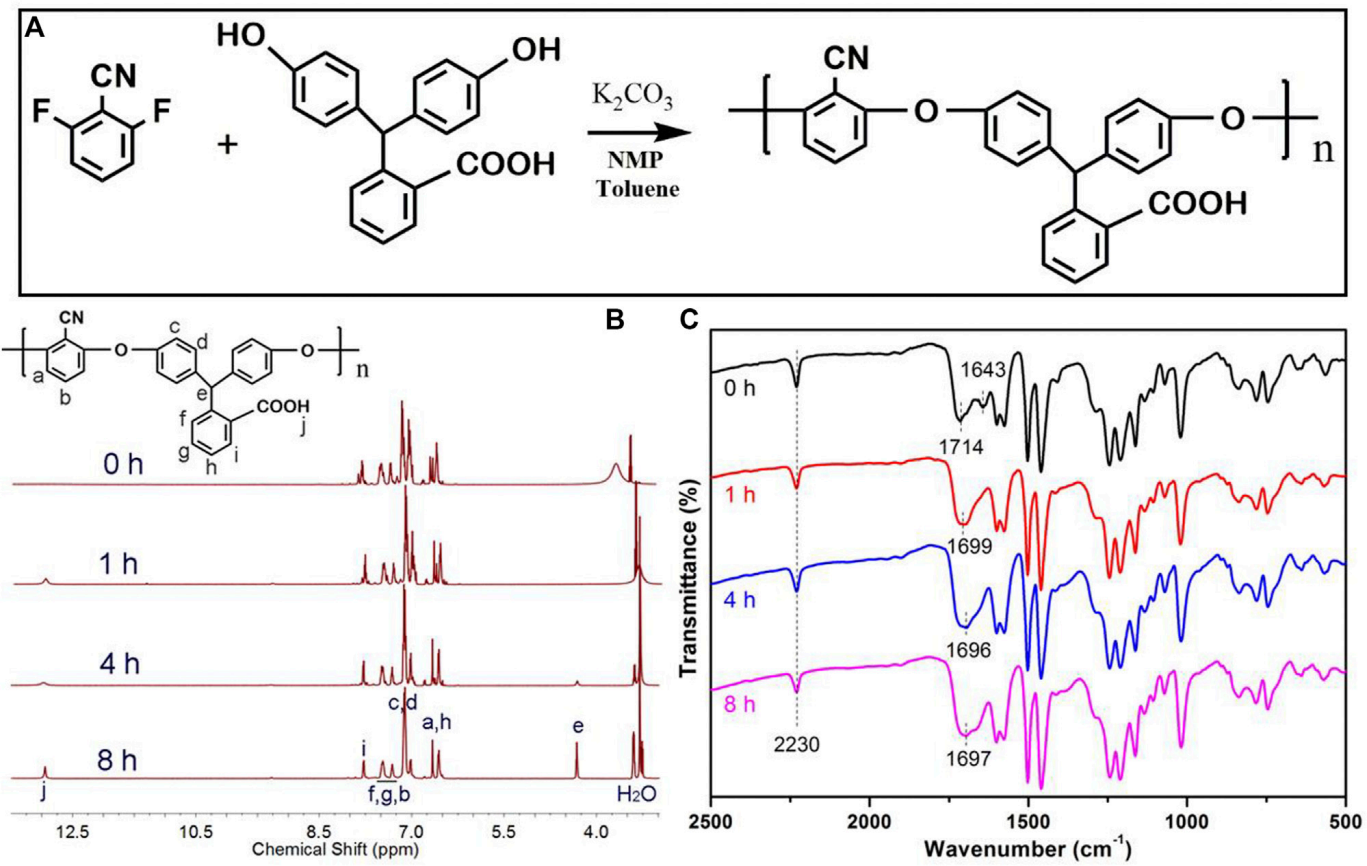
The synthetic route **(A)** for super-engineering polyarylene ether containing pendent nitrile and carboxylate (PAE-NC), 1H NMR spectra **(B)**, and FTIR spectra **(C)** of synthesized PAE-NC and PAE-NC subject to alkaline hydrolysis for a different time.

Super-engineering thermoplastics are a kind of high-performance polymers that exhibit good thermal stability and mechanical properties due to their aromatic backbone structure as well as strong intermolecular entanglements (Park et al., 2019; Wang and Zhang, 2019; Lin et al., 2022). However, the post-modification of these high-performance polymers is quite a challenge but is indispensable for many applied scenarios (Guan et al., 2019; Boydston et al., 2020; Lu et al., 2021). For instance, the interfacial compatibility between polymer matrix and inorganic nanoparticles plays a decisive role in determining the optical, magnetic, and electric functionalities of these polymeric nanocomposites (Jia et al., 2016; Liu et al., 2022; Zhang et al., 2022), which normally requires the appropriate interfacial modification on either polymer matrix or inorganic nanoparticles. In this sense, we have designed a reactive super-engineering polymer that contained pendent carboxyl and cyano groups, which exhibited versatile reactivity toward many chemical groups of inorganic nanoparticles. Specifically, the PAE-NC was synthesized using DFBN and PPL as monomers *via* the nucleophilic substitution polycondensation that can be easily scaled up according to Figure 1A. In addition, we attempted to transfer the pendent nitrile of PAE-NC into reactive groups, since the alkaline hydrolysis of nitrile leads to the generation of various reactive groups (Eloy and Lenaers, 1962; Yanaranop et al., 2016; He et al., 2021a). According to the 1H NMR spectra in Figure 1B, the PAE-NC was successfully synthesized and its chemical structure was steadily modulated along with hydrolysis treatment. Moreover, the characteristic carbonyl peak of as-synthesized PAE-NC was shifted from 1,714 cm^−1^ to around 1,696–1,699 cm^−1^ after alkaline hydrolysis according to FTIR spectra shown in Figure 1C, which could be due to the alternation of intermolecular hydrogen bonding by hydrolysis treatment. Moreover, it should be noted that the vibration peak of the nitrile group at 2,230 cm^−1^ still presented even after hydrolysis for 8 h, implying that it was quite challenging to realize the complete transformation of nitriles by the heterogeneous alkaline treatment, and similar results were also reported by another group (Sun et al., 2022). Anyway, the hydrolysis leads to partial nitriles of PAE-NC transformation into carboxyl groups, which would provide additional reactive sites for post-functionalization or further surface modification.

**FIGURE 2:**
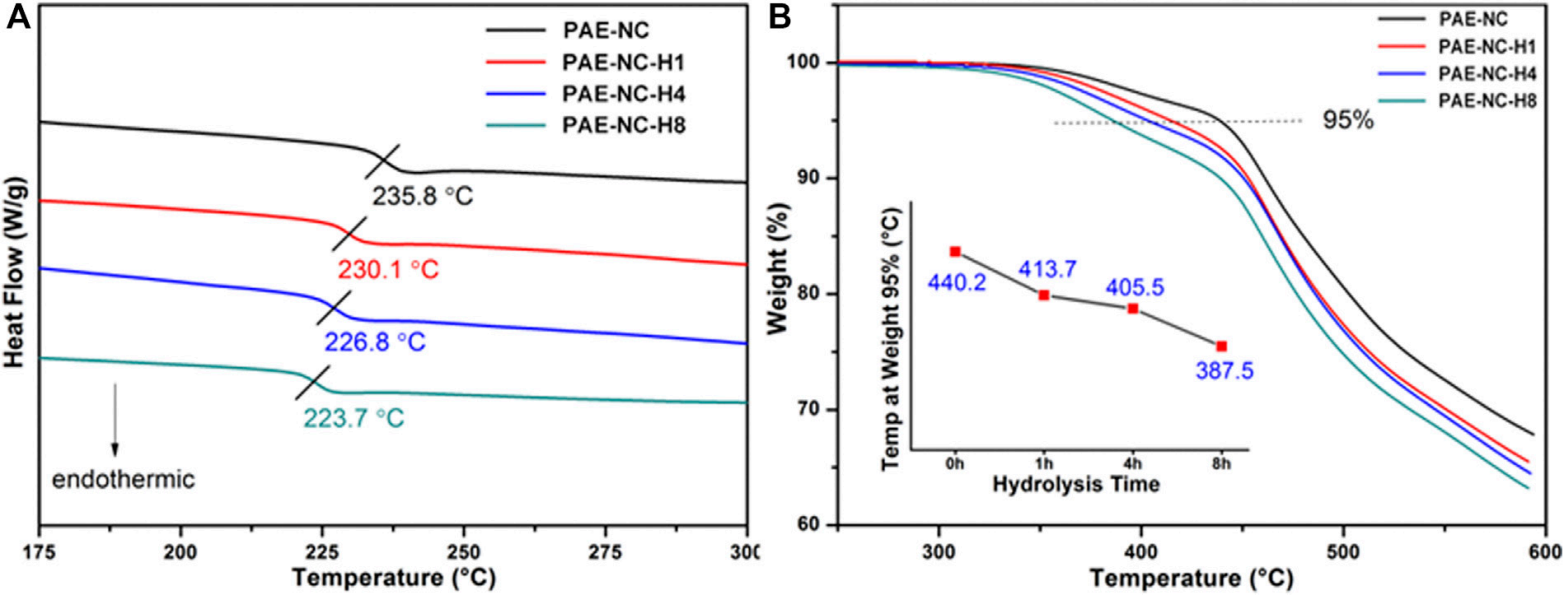
The DSC thermograms **(A)** and TGA curves **(B)** PAE-NC are subject to alkaline hydrolysis at different times.

Next, the thermal properties of as-synthesized PAE-NC and hydrolyzed PAE-NC-Hx were characterized. According to Figure 2A, the glass transition temperature of PAE-NC was gradually decreased along with the increase in hydrolysis time, which was supposed to be attributed to the attenuated hydrogen bonding between nitrile and carboxyl during prolonged hydrolysis (Le Questel et al., 2000). Moreover, the TGA results in Figure 2B also exhibited decreased thermal stability with increasing hydrolysis treatment, as the decomposition temperature at 5% was decreased from 440.2°C for as-synthesized PAE-NC to 387°C after hydrolysis for 8 h. Despite slightly decreased glass transition temperature and decomposition temperature, the hydrolyzed PAE derivatives still exhibited good stability up to 350°C, which was comparable to many commercially available super-engineering polymers including polyether ether ketone (PEEK), polyethersulfone (PES), and polyphenylene sulfide (PPS).

**FIGURE 3:**
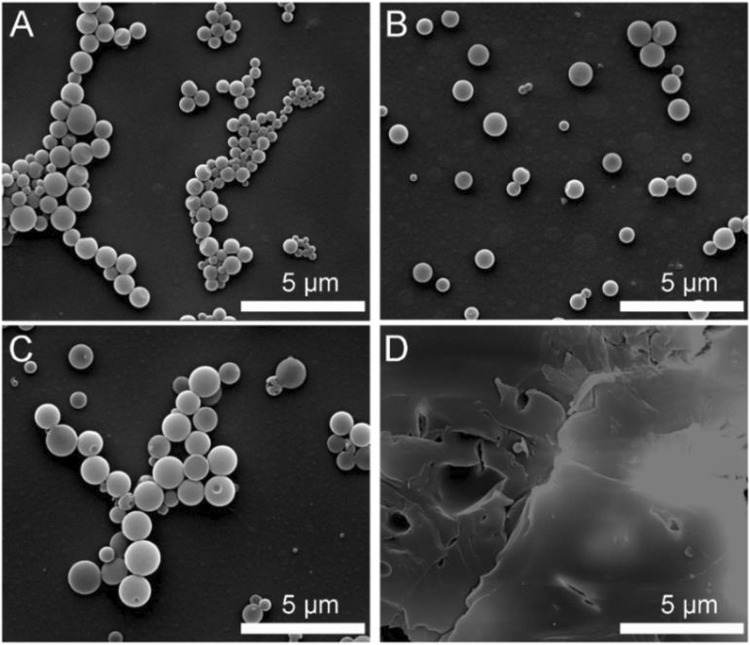
The SEM images of polymeric microparticles prepared by using pristine PAE-NC **(A)** and PAE-NC after alkaline hydrolysis for 1 h **(B)**, 4 h **(C)**, and 8 h **(D)**, respectively.

Furthermore, the surface morphology of obtained polymeric microparticles was characterized by SEM. According to Figure 3A, the polymeric microparticles with invaginate pit morphology were obtained from the PAE-NC, and the polymeric microparticles of larger average size with a smoother surface (Figure 3B) or smaller pinhole morphology (Figure 3C) were detected from the sample obtained using PAE-NC-H1 and PAE-NC-H4, respectively. The average size information of prepared superparticles was calculated and listed in Table 1. Interestingly, the irregular membrane structures without any microparticles were observed from the PAE-NC-H8 sample (see Figure 3D), which should be attributed to the declined hydrophobic interaction derived from the enhanced water solubility of this sample after a long time of hydrolysis.

**TABLE 1:** The average diameters of pristine and QD@PAE hybrid microparticles.

Polymer matrix	PAE-NC	PAE-NC-H1	PAE-NC-H4
Pristine polymer particles	390 ± 17 nm	610 ± 32 nm	851 ± 20 nm
QD@PAE hybrid particles	430 ± 30 nm	750 ± 29 nm	960 ± 46 nm

**FIGURE 4:**
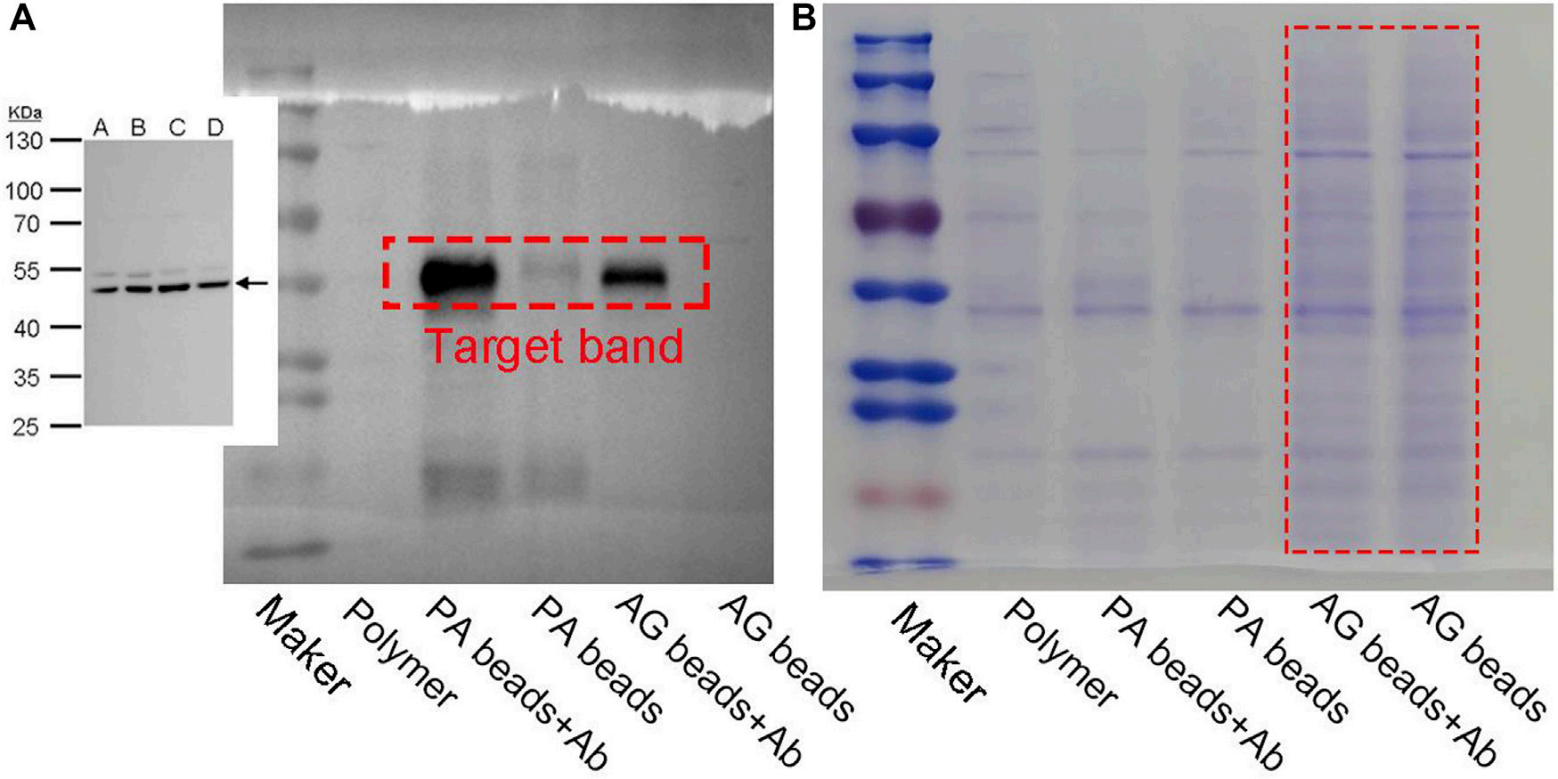
The Western blot **(A)** and Coomassie blue staining **(B)** plot of protein enrichment using protein A modified PEN-NC microparticles (AP beads) and commercially available AG beads.

Since the polymer beads obtained from PAE-NC-H1 exhibited a more uniform size and smoother surface, they were selected as the optimized sample for constructing post-functionalized beads. As a proof-of-concept application, the PAE-NC-H1 polymer was first converted to pristine polymer beads *via* emulsion confinement self-assembly, then the surface carboxyl groups of self-assembled polymer beads were activated with an EDC agent (see details in experimental section 2.4), followed by covalent immobilization of protein A *via* classical amidation reaction to obtain bio-functionalized beads (abbreviated as PA beads). Due to the specific antibody affinity properties of protein A, the PA beads could serve as bio-separation scaffolds to enable antibody enrichment from complex bio-fluids. In addition, we also compared the antibody enrichment performance of as-prepared PA beads and commercially available agarose beads (AG in short) from cell lysis solution. It was clear that when compared with the commercial AG beads, our PA beads exhibited improved antibody enrichment and lower non-specific binding effect according to the Western blot assay results in Figure 4A and Coomassie blue staining results in Figure 4B, respectively.

**FIGURE 5:**
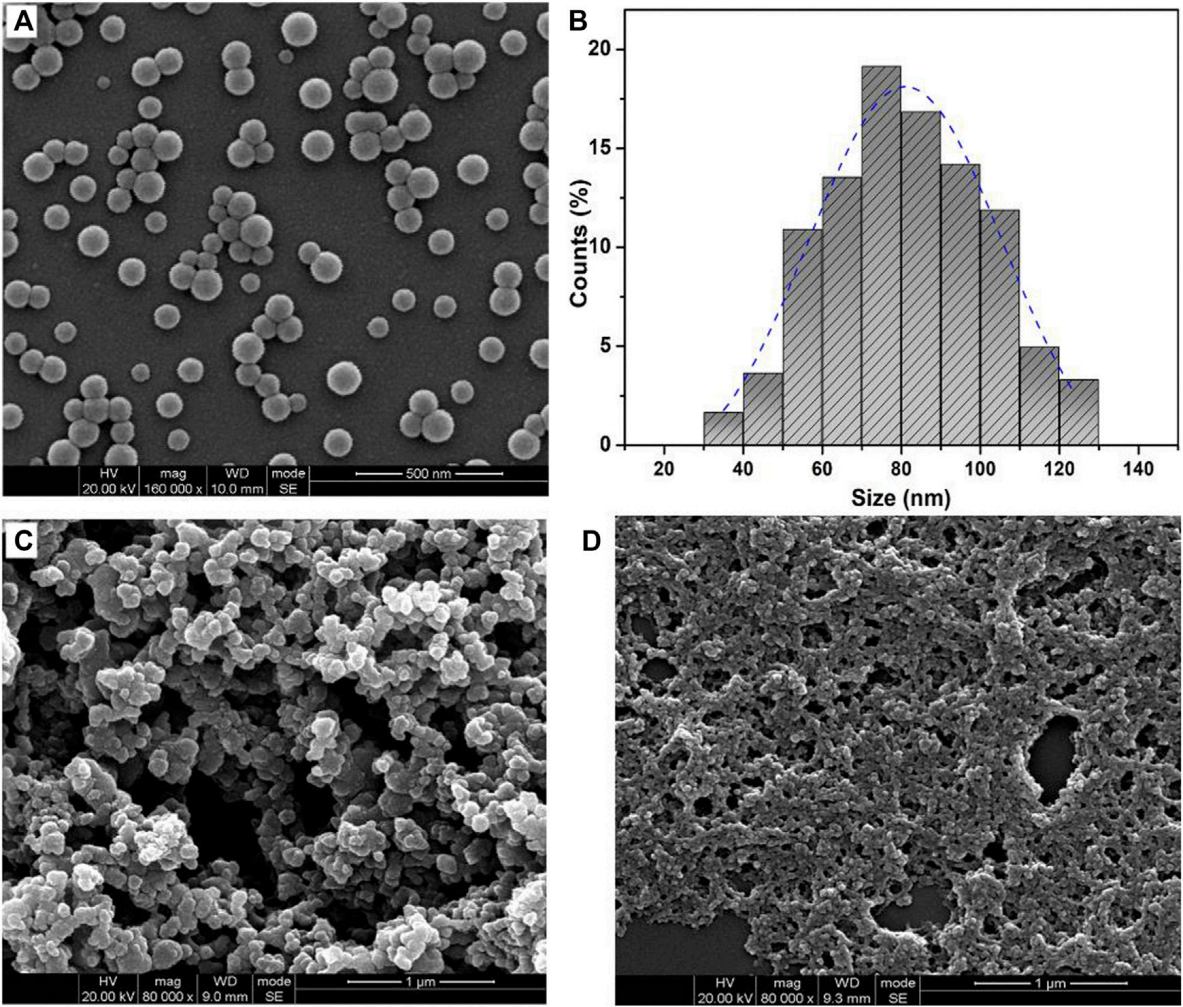
The SEM morphology **(A)** and corresponding size distribution histogram **(B)** of polymeric nanoparticles obtained *via* the solvent exchange–induced self-assembly of pristine PAE-NC, and the morphology of Zn^2+^ **(C)** and Cu^2+^ **(D)** cross-linked PAE-NC self-assembled nanostructures.

In addition to emulsion confinement self-assembly, we also prepared the self-assembled PAE-NC structures of different morphology using classical solvent exchange protocol. More specifically, the solvent exchange–induced self-assembly of PAE-NC was conducted by rapid injection of polymer solution in good solvent to its non-solvents system. It should be observed that much smaller-sized polymeric nanoparticles were obtained by introducing PEN solution in DMF into mixed solvents of toluene and ethanol (volume ratio of 1:2) according to Figure 5A, and the average diameter was determined to be 81 ± 1.2 nm according to Figure 5B. In addition, we also found that the metal ions crosslinking with carboxyl groups of PAE-NC had an obvious influence on the surface morphology of solvent exchange–induced self-assembled structures. Larger-sized necklace-like aggregates were observed from the Zn^2+^ cross-linked sample (see Figure 5C), while much smaller nanoparticle networks were generated after adding Cu^2+^ to the self-assembly of PAE-NC according to Figure 5D. The totally different sizes of resultant particles between solvent exchange–induced self-assembly and emulsion confinement self-assembly should be attributed to the different driven forces and mechanisms.

**FIGURE 6:**
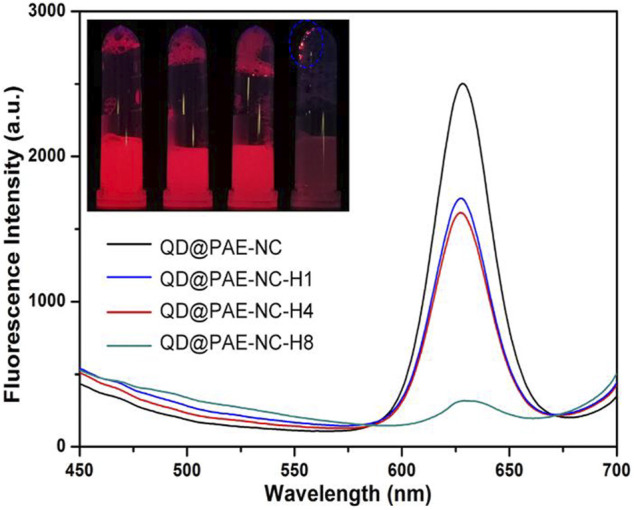
The fluorescent emission spectra from the aqueous solution containing various QD@PAE-NC composite microparticles; inset image was the samples photo under UV light.

Considering that both the as-synthesized PAE-NC and its hydrolyzed derivatives can be self-assembled into microparticles, the process could also be employed for encapsulation of vulnerable compounds or inorganic nanoparticles to preserve their functionalities. With this regard, the commercially available highly fluorescent quantum dots (QDs) of ZnCdSe@ZnS stabilized by the hydrophobic surface ligand of oleic acid were used as an example to evaluate the encapsulation capacity of PAE-NC matrix, as the typical red emission of hydrophobic QD could be easily quenched by water without appropriate surface modification (Pellegrino et al., 2004; He et al., 2021b). Figure 6 demonstrated the fluorescent emission spectra recorded from the aqueous solution of QD@PAE-NC composites. It was clear that the red emission peak at 627 nm of QD can be well-preserved after being encapsulated in PAE-NC microparticles. Moreover, the PAE-NC-H1 and PAE-NC-H4 also exhibited good encapsulation of QD, while the composites involving PAE-NC-H8 showed a limited encapsulation effect in terms of preserving QD fluorescence. Moreover, the variation of fluorescence emission from these QD@PAE samples was also confirmed by the inset photo.

**FIGURE 7:**
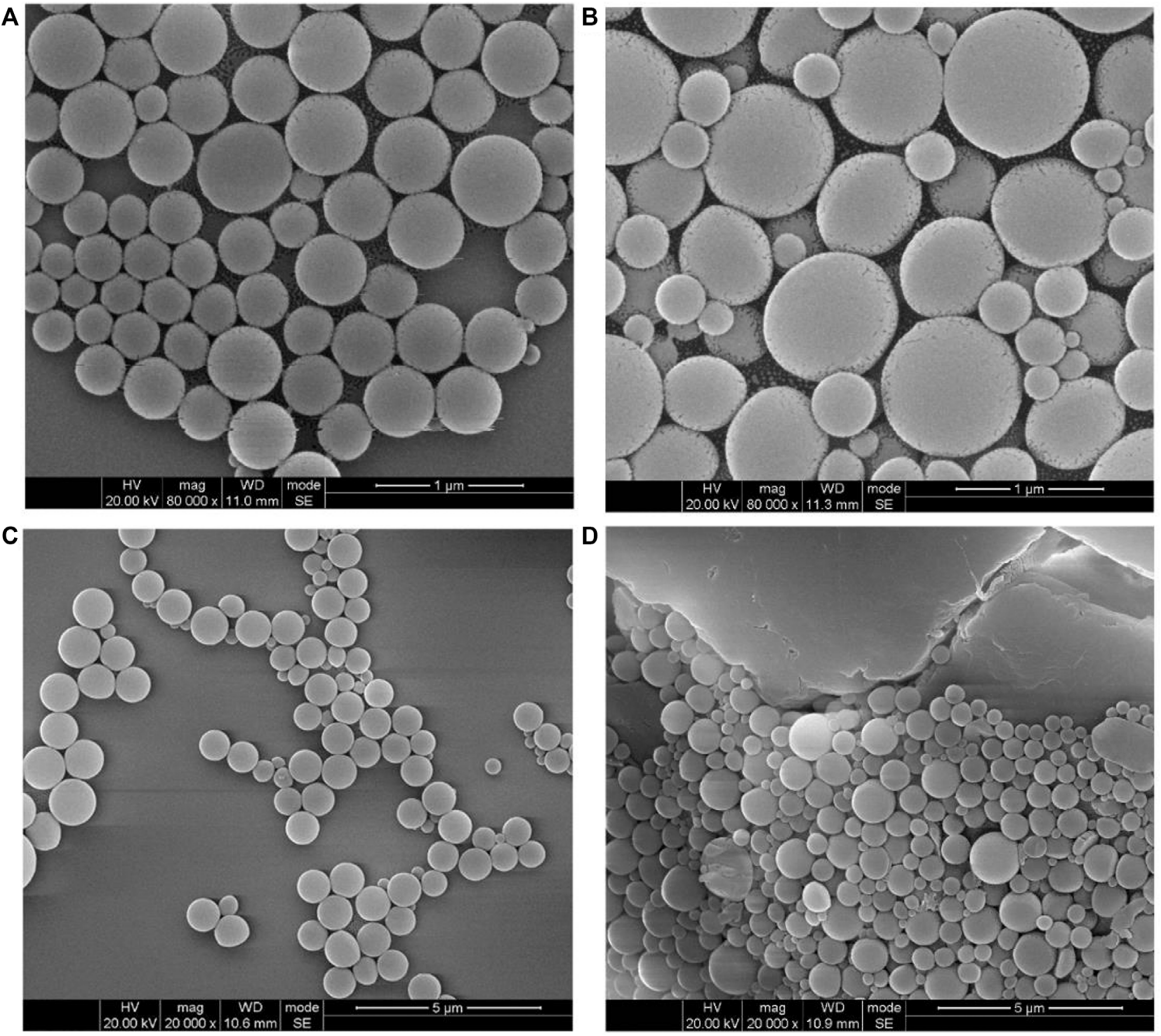
The SEM morphology of QD-embedded polymeric hybrid particles using pristine PAE-NC **(A)**, hydrolyzed PAE-NC-H1 **(B)**, hydrolyzed PAE-NC-H4 **(C)**, and PAE-NC-H8 **(D)**, respectively.

The surface morphology characterization also proved that the composite beads using pristine PAE-NC (Figure 7A), PAE-NC-H1 (Figure 7B), and PAE-NC-H4 (Figure 7C) showed similar morphology, and their average diameter data were summarized in Table 1 as well. Lastly, the microparticles mixed with continuous membranes were observed from sample PAE-NC-H8 (Figure 7D). The different encapsulation capacity and surface morphology would be due to the attenuation of hydrophobic interaction between PAE-NC and surface ligand of QD along with increased hydrolysis treatment. It should be observed that the hydrophobic nanoparticles encapsulation capacity of PAE-NC was also applicable for other fluorescent quantum dots or even magnetic nanoparticles.

## 4 Conclusion

In this work, we have synthesized a super-engineering thermoplastic of PAE-NC containing pendent reactive groups. Furthermore, we demonstrated that both as-synthesized PAE-NC and its hydrolysis derivatives can be self-assembled into polymeric microparticles. The size and fine morphology of these polymeric microparticles can be easily modulated by using different self-assembly protocols or crosslinking metal ions. In addition, the prepared polymeric microparticles can be rendered with additional bio-separation functionality after post-biomodification with the biorecognition agent of protein A. Moreover, the emulsion confinement self-assembly of PAE-NC and its hydrolyzed derivatives can be employed to encapsulate hydrophobic QD and preserve its red fluorescent in an aqueous solution. It should be observed that although the encapsulation capacity of PAE-NC was evaluated using fluorescent QD for the moment, the protocol revealed in the current work is applicable to other hydrophobic functional nanoparticles. Considering the high thermal stability, abundant surface reactive groups, and encapsulation properties of PAE-NC microparticles, the current work would open a new way to fabricate microparticles or microcapsules to preserve the functionalization of various sensitive or hazardous components including explosive, radioactive agents.

## Data Availability

The original contributions presented in the study are included in the article/Supplementary Material; further inquiries can be directed to the corresponding author.
